# Sacrocolpopexy Using Polyvinylidene Fluoride Mesh: A Systematic Review and Meta‐Analysis

**DOI:** 10.1155/aiu/1620419

**Published:** 2026-06-12

**Authors:** Mahdi Norouzi, Mahtab Zargham, Farshad Gholipour, Shayan Ghanouni, Raheleh Karimi, Farshid Alizadeh

**Affiliations:** ^1^ Isfahan Kidney Disease Research Center, Isfahan University of Medical Sciences, Isfahan, Iran, mui.ac.ir; ^2^ School of Medicine, Isfahan University of Medical Sciences, Isfahan, Iran, mui.ac.ir; ^3^ Department of Urology, School of Medicine, Isfahan University of Medical Sciences, Isfahan, Iran, mui.ac.ir; ^4^ Department of Epidemiology and Biostatistics, Isfahan University of Medical Sciences, Isfahan, Iran, mui.ac.ir; ^5^ Department of Urology, West Virginia University, Morgantown, West Virginia, USA, wvu.edu

**Keywords:** pelvic organ prolapse, polypropylene, polyvinylidene fluoride, sacrocolpopexy, synthetic mesh

## Abstract

**Background and Aims:**

This review aimed to assess the clinical outcomes and complications of polyvinylidene fluoride (PVDF) mesh in sacrocolpopexy (SCP).

**Methods:**

A systematic search was conducted on Google Scholar, PubMed, Web of Science, Scopus, and Embase up to October 2024. All study designs employing PVDF mesh in SCP were included. Demographic features, mesh types, surgical method, surgical outcomes, and mesh exposure, among other outcomes and complications, were extracted by two independent researchers. The registered protocol is available online on PROSPERO (ID: CRD42023405734).

**Results:**

Out of 319 studies screened, 12 were finally included in the systematic review and meta‐analysis, encompassing 1093 female patients who underwent SCP. Four studies employed polypropylene (PP) as the comparator. Abdominal sacrocolpopexy (ASC) was used by six studies, five adhered to the laparoscopic method and one used both. Meta‐analysis indicated pooled prevalence rates close to 0% for mesh exposure, recurrence, sexual dysfunction (SD), and reoperation in the PVDF group; however, small sample sizes and wide confidence intervals diminish the definitive conclusion. The prevalence of urge urinary incontinence (UUI) was 33%, while both stress urinary incontinence (SUI) and *de novo* incontinence had a prevalence of 6%. However, the differences between PVDF and PP outcomes and complications were not statistically significant.

**Conclusion:**

No significant differences were found between PVDF mesh and PP. Our findings highlight the need for further robust evidence to indicate the possible differences between PVDF and conventional materials, especially PP.

## 1. Introduction

Approximately 30% of US females aged between 50 and 89 years old are affected by pelvic organ prolapse (POP), a condition characterized by the anatomical disposition of the pelvic organs and bulging through the vagina [1]. The prevalence of POP is expected to rise progressively, even up to 50% by 2050 [2]. POP significantly affects women’s quality of life [3]. About 73% of POP patients suffer from urine incontinence, 86% from urinary urgency, 34%–62% from voiding dysfunction, and 31% suffer from fecal incontinence (according to integral theory) [4].

Open abdominal sacrocolpopexy (ASC), introduced by Lane in 1962 [5], remains the gold standard procedure for high‐grade apical POP repair [6]. However, recent studies encompassing laparoscopic and robotic procedures reflect relatively superior functional and anatomical outcomes [7, 8]. Sacrocolpopexy (SCP), according to Cochrane, has demonstrated superior long‐term outcomes compared with vaginal sacrospinous ligament fixation although it requires longer operative and recovery times [9]. Meta‐analysis comparing SCP with transvaginal mesh surgery found lower mesh‐related complication rates, prolapse recurrence, and de novo dyspareunia. Nonetheless, side effects are inevitable: reoperation rates range from 2.3% in clinical studies [10] to 29.2% in large administrative datasets [11]. A systematic review including 71 RCTs indicated 110 different complications of the procedure, with erosion (78% of studies), pain (56%), bleeding (61%), and dyspareunia (49%) being the most common ones [12].

Polyvinylidene fluoride (PVDF) mesh, first introduced in 2002 [13], has emerged as an alternative to polypropylene (PP) [14]. A histological study on sheep showed higher biocompatibility of PVDF compared with PP [15]. Additionally, PVDF has shown no sign of degradation phenomenon (cracking and flaking on the surface of mesh), a growing concern in long‐term mesh safety, even after 24 months of follow‐up [16]. Contrary to PVDF, PP showed progressive degradation at the same time, which may account for the corresponding privileges [17]. The present study aims to systematically review and meta‐analyze the current literature on SCP using PVDF mesh to provide robust evidence on the clinical application of PVDF.

## 2. Methods

### 2.1. Search Strategy

Google Scholar, PubMed (MEDLINE), Web of Science, Scopus, and Embase were systematically searched up to October 2024 using the following search line: ((“Sacrocolpopexy” OR “Sacral colpopexy”) AND (“PVDF” OR “Polyvinylidene fluoride”)). The search strategy was tailored to each of the databases (detailed search string for each of the databases is available in the supporting materials). Studies were then restricted to the English language, and duplicates were deleted. No time limitation was considered. This review was conducted following the 2020 version of Preferred Reporting Items for Systematic Reviews and Meta‐Analyses (PRISMA) guidelines [18]. The registered protocol is available on PROSPERO (ID: CRD42023405734).

### 2.2. Inclusion and Exclusion Criteria

Followed by the search strategy, all types of studies on POP repair using SCP and PVDF mesh were included without restrictions on time, region, or age. Only human studies were included. Exclusion criteria were as follows: non‐English studies, review articles, case reports, conference abstracts, and studies using multiple mesh types without adequate details.

### 2.3. Study Selection and Data Extraction

Studies were screened and extracted by two independent researchers (MN and FGH), and controversies were solved through mutual discussion. The data extraction items were as follows: first author, country, publication date, study design, participants, sex, age, duration of follow‐up, mesh type, operation features, surgical procedure, pre‐ and postoperative data, anatomical and functional outcomes, mesh exposure, and other side effects (when applicable). Discrepancies regarding study selection, data extraction, analyses, and risk of bias (ROB) assessment were resolved through joint discussion.

### 2.4. Quality Assessment

ROB assessment was conducted by two independent researchers, and any disagreements were resolved through discussion. We used the Newcastle–Ottawa Scale (NOS) tool. Risk assessment categories encompass selection bias, comparability, exposure (for case controls), and outcome (for cohort/cross sections). Using the scores from each category, the overall score was computed, and studies were then categorized into low risk, unclear, or high risk based on their respective scores.

### 2.5. Statistical Analysis

Statistical analysis was conducted using STATA 16.0 (StataCorp, College Station, TX, USA). Descriptive statistics were used to summarize study characteristics. Continuous variables were expressed as mean ± standard deviation (SD) and categorical variables as frequency (*n*) and percentage (%). Meta‐analysis was used for both, pooling the prevalence of PVDF outcomes and comparing the outcomes of PVDF versus PP. For analysis with more than 10 studies, publication bias was assessed visually via funnel plots and, where applicable, statistically via Egger’s regression test [19]. In the prevalence meta‐analysis, the pre‐ and postoperative incidence of each outcome was either extracted or calculated and used for meta‐analysis. Incidence of the functional and anatomical outcomes was compared with the respective preoperative incidence. For the *de novo* incontinence outcome, the incidence was compared with the crude baseline participants of each study. All statistical tests were two sided, and a *p* value < 0.05 was considered statistically significant. ORs are presented with 95% confidence intervals (CIs).

## 3. Results

### 3.1. Study Selection

**FIGURE 1:**
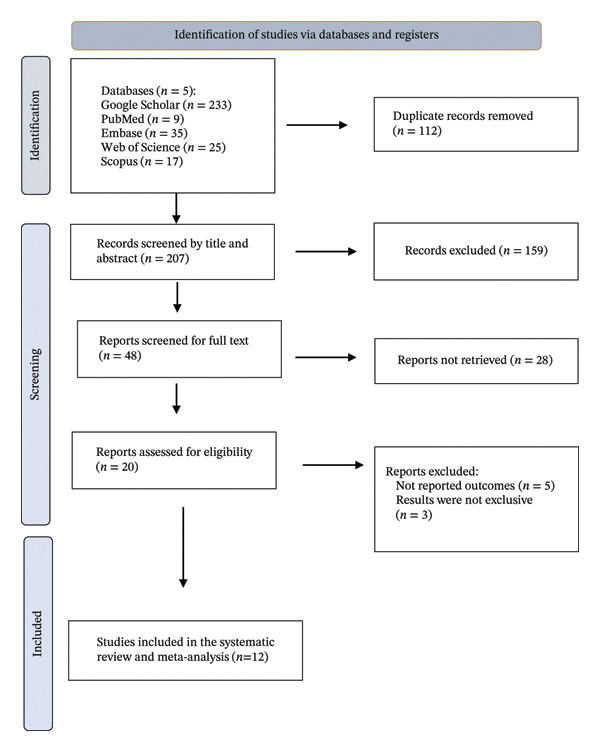
PRISMA flowchart of included studies in systematic review and meta‐analysis.

The study selection process is summarized in Figure 1. Based on the search strategy, 363 records were identified, and after excluding duplicates, 242 papers remained for further screening. Titles and abstracts were carefully screened by two researchers, and 48 papers underwent full‐text screening. Finally, 12 studies were included in the systematic review and meta‐analysis.

### 3.2. Characteristics of Included Studies

Table 1 summarizes the characteristics of the included studies. Of 12 studies, 10 were cohort [14, 20–28], one was an RCT [29] and 1 was a case–control [30]. The publication year ranged between 2015 and 2023 [14, 24]. Mean follow‐up duration was 50.9 months for PVDF and 53 months for PP, ranging between 4 and 410 months [27, 29]. Overall, 1093 female patients who underwent SCP were included. The largest study was conducted on 210 females, while the smallest encompassed 10 females [20, 23]. Six studies used ASC [14, 21, 24, 25, 28, 29], five used laparoscopic procedures [20, 22, 23, 26, 27], and one used both [30]. Four studies used PP mesh as the PVDF comparator. The main outcomes of the studies were functional and anatomical and mesh complications.

**TABLE 1:** Characteristics of included studies.

Study	Region	Design	Population	Age (mean, year)	Mesh	Follow‐up (months)	Procedure
Balsamo et al., [30]	Italy	Retrospective case‐control	136 (PVDF: 63, PP: 73)	PP: 68.97, PVDF: 67.96	PVDF^∗^ vs. PP^∗∗^	PVDF: 25.6, PP: 94	Laparoscopic or open
Joukhadar et al., [20]	Germany	cohort	10	62	PVDF	12	Laparoscopic
Rajshekhar et al., [21]	UK	Retrospective cohort	50	66	PVDF	13	Open
Kavallaris et al., [22]	Greece	Retrospective cohort	172 (PVDF: 90, PP: 82)	PVDF: 59.5, PP: 61.2	PVDF vs. PP	PVDF: 41, PP: 54	Laparoscopic
Callewaert et al., [23]	Belgium	Cohort	210 (PVDF = 105, PP = 105)	PVDF: 67, PP: 68	PVDF vs. PP + PG	PVDF: 26, PP: 46	Laparoscopic
Jaeger et al., [24]	Germany	Cohort	76	64.9	PVDF	20	Open
Cassis et al., [25]	UK	Retrospective cohort	100	65.2	PVDF	12	Open
Ludwig et al., [28]	Germany	Retrospective cohort	71	64	PVDF	16	Open
Eisenberg et al., [26]	Israel	Retrospective cohort	42 (PVDF: 25, PP: 17)	PVDF: 62.16, PP: 56.82	PVDF vs. PP	18	Laparoscopic
de Castro et al., [29]	Brazil	RCT	71	64.5	PVDF	410.2	Open
Rexhepi et al., [27]	Germany	cohort	120	66	PVDF	4	Laparoscopic
Zargham et al., [14]	Iran	cohort	35	59.9	PVDF	12	Open

^∗^PVDF: polyvinylidene fluoride.

^∗∗^PP: polypropylene.

### 3.3. Synthesis of Results

Functional outcomes of SCP were categorized and analyzed in six categories: stress urinary incontinence (SUI), urge urinary incontinence (UUI), storage symptoms (SS), voiding symptoms (VS), sexual dysfunction (SD), and dysfunctions occurring postoperation (*de novo* urinary incontinence).

### 3.4. Prevalence of Outcomes

Table 2 presents a summary of the results included in the prevalence meta‐analysis. The pooled meta‐analysis showed the prevalence of mesh extrusion as 0% (Figure S1). Likely, the pooled prevalence of POP recurrence, SD, and reoperation was 0% (Figures S2, S3, and S4). Persistence of UUI after the operation, with a pooled prevalence of 33%, was the most prevalent postoperative complication (Figure S5). Both SUI and *de novo* incontinence had a prevalence of 6% among patients (Figures S6 and S7). Publication bias assessment using funnel plots and Egger’s regression test was performed for mesh extrusion and reoperation prevalence (with 10+ studies). Funnel plots are presented in Figures S8 and S9. Egger’s test revealed no evidence of small study effects (i.e., publication bias) for mesh extrusion (*p* = 0.33) and reoperation (*p* = 0.24).

**TABLE 2:** Incidence of PVDF mesh outcomes.

Study	Mesh erosion (%)	Recurrence (%)	Reoperation (%)	De novo urinary incontinence (%)	Stress urinary incontinence (%)	Urge urinary incontinence (%)	Sexual dysfunction (%)	Storage symptoms (%)	Voiding symptoms (%)
Balsamo et al., [30]	1.6	14.3	0	3.1	20.7	71.4	0	0	13
Kavallaris et al., [22]	0	0	0	7.8	39.1	18.4	4.3	3.1	0
Callewaert et al., [23]	1.9		1.9		32.1	41.1	50		
Rajshekhar et al., [21]	0	2	2	8	9		0		
Joukhadar et al., [20]	0	10	0	10	16.6				
Rexhepi et al., [27]	0				30.7				
Zargham et al., [14]	0	17.1	5.7	5.7					
de Castro et al., [29]	5.6	32.4	7						
Jaeger et al., [24]	0	6.6	6.6						
Cassis et al., [25]	2	12	4						
Eisenberg et al., [26]	0	0	0						
Ludwig et al., [28]	0								

## 4. PVDF vs. PP

### 4.1. Functional Outcomes

**FIGURE 2:**
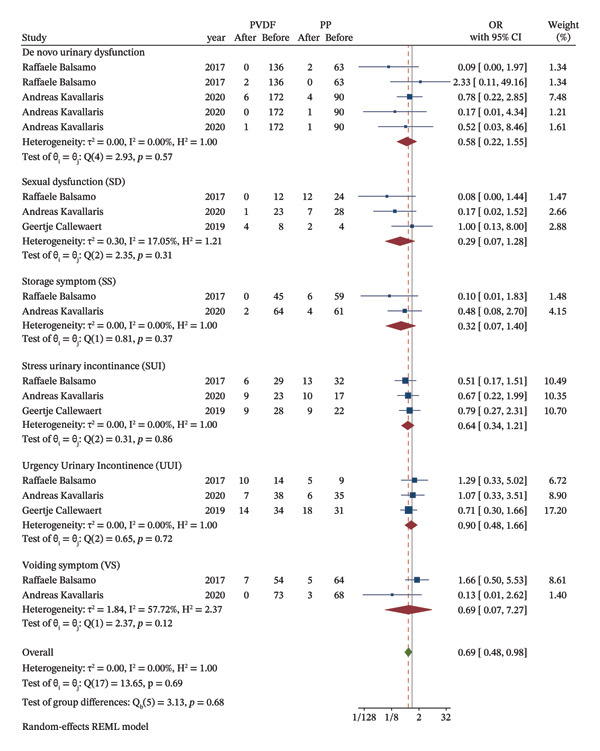
Forest plot of meta‐analysis and subgroup meta‐analysis of PVDF vs. PP outcomes.

SUI: SUI was reported in three studies. Although the incidence of SUI after SCP with PVDF was lower than PP, the results were not statistically significant (OR = 0.64 [CI 95%: 0.34, 1.21], *p* = 0.86) (Figure 2).

UUI: Similar to SUI, UUI was reported in 3 studies. Thirty‐six percent of all patients in the PVDF group and 38.6% of the PP group had UUI symptoms after surgery. However, the differences between groups were not statistically significant (OR = 0.90 [CI 95%: 0.48, 1.66], *p* = 0.72) (Figure 2).

SD: Although the rates of SD after surgery were lower in the PVDF group than in PP, the results did not show a statistically significant difference (OR = 0.29 [CI 95%: 0.07, 1.28], *p* = 0.31) (Figure 2).

SS: SS was reported in two studies, and rates were lower for PVDF compared with PP in both studies. However, the differences were not statistically significant (OR = 0.32 (0.07, 1.40), *p* = 0.37) (Figure 2).

VS: Persistence of VS after surgery was reported in two studies, which reported conflicting results. The differences were not statistically significant (OR = 0.69 [CI 95%: 0.48, 1.66], *p* = 0.72) (Figure 2).

*De novo* urinary dysfunctions were defined as new occurrences of urinary dysfunctions postoperatively, which were reported by two studies. The overall differences between PVDF and PP were not statistically significant (OR = 0.58 [CI 95%: 0.22, 1.55], *p* = 0.57) (Figure 2).

The overall differences between the functional outcomes of PVDF compared with PP were not statistically significant (OR = 0.69 [CI 95%: 0.48, 0.98], *p* = 0.68) (Figure 2).

### 4.2. Mesh Exposure

**FIGURE 3:**
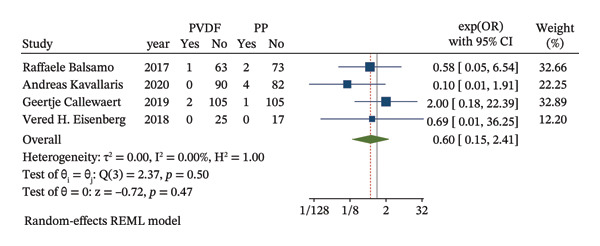
Forest plot of PVDF vs. PP mesh extrusion.

Out of four studies that reported mesh complications in both PVDF and PP, three studies indicated a lower incidence of exposure in PVDF compared with PP. The crude incidence of exposure was 1% and 2.5% in the PVDF vs. PP group, respectively. However, the results did not show a statistically significant difference (OR = 0.60 (0.15, 2.41), *p* = 0.47) (Figure 3).

### 4.3. ROB Assessment

**FIGURE 4:**
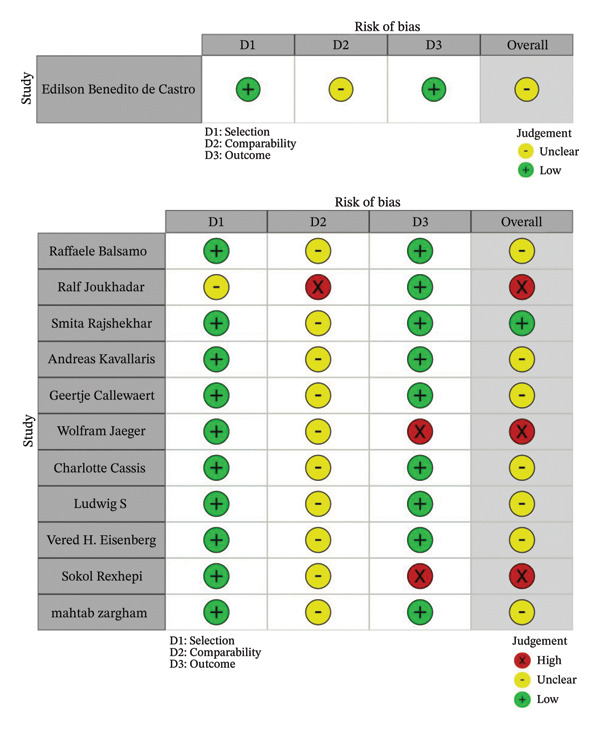
Summary of risk of bias assessment for RCT (the upper table) and cohort studies (the lower table).

**FIGURE 5:**
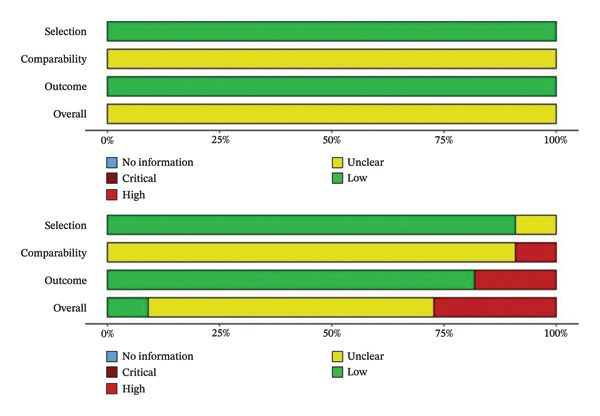
Risk of bias graphs for (a) RCT and (b) cohort studies.

The ROB assessment was conducted using the NOS tool and is illustrated in Figures 4 and 5 [31]. Concerns regarding comparability affected all studies. Most studies on other categories indicated good performance.

## 5. Discussion

The present systematic review aimed to investigate the outcomes of SCP using PVDF mesh in the treatment of POP and compare the outcomes with PP mesh as an auxiliary investigation. However, there were no statistically significant differences between the two mesh types. Further studies are required to elucidate the differences between mesh types.

Various factors, such as concomitant procedures, surgical approach, surgeon experience, and patients’ conditions, can contribute to complications and may significantly influence outcomes. The inability to control these confounding factors in our study may have impacted the observed results. Additionally, most of the included studies were observational, which is prone to potential confounding by indication. For instance, surgeon preferences, complexity of the surgical method, or patients’ characteristics are influential factors in choosing mesh material and surgical endpoints. Although some studies highlight the advantages of PVDF over PP, statistical significance was not achieved in our meta‐analysis. Although meta‐analysis indicated a pooled prevalence of 0% for mesh extrusion, recurrence, reoperation, and SD, the results were not statistically significant compared with PP. Additionally, the small number of included studies and wide CIs diminish the strong conclusion on this matter; further robust studies are required to draw more confident evidence. On the other hand, these procedures were performed by experienced surgeons in advanced centers, which may limit the generalizability. Similar to our study, a systematic review on PVDF vs. PP mesh types in POP/UI encompassing three studies on POP surgery reported no differences between mesh outcomes, except for *de novo* urgency and *de novo* sexual dysfunction; however, this significant difference was not replicated in our study [32].

Among 816 PVDF patients with a mean follow‐up of 50.9 months, only nine (1.1%) experienced mesh extrusion, a rate lower than what has been reported in other reviews for synthetic mesh extrusion [33]. Additionally, one superiority of PVDF compared with PP is radiologic visibility. Kavallaris and Zygouris [22] reported PVDF’s privilege in the early diagnosis of intraoperative injuries through MRI visibility. Unlike PVDF, PP mesh is not easily visualized on imaging modalities.

Several limitations affected our study. First, small sample sizes reduce the statistical power and robustness of the outcomes. Second, the short to moderate follow‐up durations in available studies restrict the ability to assess long‐term safety and durability of PVDF mesh in POP repair. Third, there was substantial heterogeneity in outcome definitions, surgical techniques, and reporting standards, which complicates direct comparisons and meta‐analytic synthesis. Fourth, the lack of adjustment for potential confounding factors—such as patient age, comorbidities, prolapse stage, and surgeon experience—may have influenced the reported outcomes. Despite the limitations, the present study highlights the relevance of mesh type selection in POP repair surgery. Furthermore, the study underscores the need for more robust studies, especially randomized controlled trials involving larger patient populations with extended follow‐up periods. Future research should compare PVDF mesh outcomes with other synthetic meshes and nonmesh approaches in POP repair.

## 6. Conclusions

In conclusion, no significant differences were found between PVDF mesh and PP. However, beyond the surgical outcomes, PVDF has shown promising results in animal studies. Also, PVDF has shown superiority in terms of mesh imaging, which is crucial in postoperation settings. Finally, we strongly recommend conducting further robust studies comparing different mesh materials in POP repair, particularly the PVDF and PP.

## Funding

No funding was received for this manuscript.

## Disclosure

All authors have read and approved the final version of the manuscript, the corresponding author has full access to all of the data in this study, and takes complete responsibility for the integrity of the data and the accuracy of the data analysis.

The corresponding author affirms that this manuscript is an honest, accurate, and transparent account of the study being reported; that no important aspects of the study have been omitted; and that any discrepancies from the study as planned (and, if relevant, registered) have been explained.

## Conflicts of Interest

The authors declare no conflicts of interest.

## Supporting Information

Additional supporting information can be found online in the Supporting Information section.

## Supporting information


**Supporting Information** Supporting File 1 (Search strings.docx): This document contains the exact search strategies applied to PubMed, Scopus, Web of Science, Embase, and Google Scholar for the systematic literature review. Figure S1. Forest plot of prevalence of mesh extrusion in PVDF patients. Figure S2. Forest plot of prevalence of prolapse recurrence in PVDF patients. Figure S3. Forest plot of prevalence of reoperation in PVDF patients. Figure S4. Forest plot of prevalence of sexual dysfunction in PVDF patients. Figure S5. Forest plot of prevalence of urge urinary incontinence in PVDF patients. Figure S6. Forest plot of prevalence of stress urinary incontinence in PVDF patients. Figure S7. Forest plot of prevalence of *de novo* urinary incontinence in PVDF patients. Figure S8. Funnel plot of mesh extrusion prevalence. Figure S9. Funnel plot of reoperation prevalence.

## Data Availability

The authors confirm that the data supporting the findings of this study are included within the article and/or its supporting materials.
