# Supplementary figures and images for: Sacrocolpopexy Using Polyvinylidene Fluoride Mesh: A Systematic Review and Meta‐Analysis
Source: Adv Urol. 2026 Jun 12;2026:1620419. doi: 10.1155/aiu/1620419 (PMC13263533; doi:10.1155/aiu/1620419)

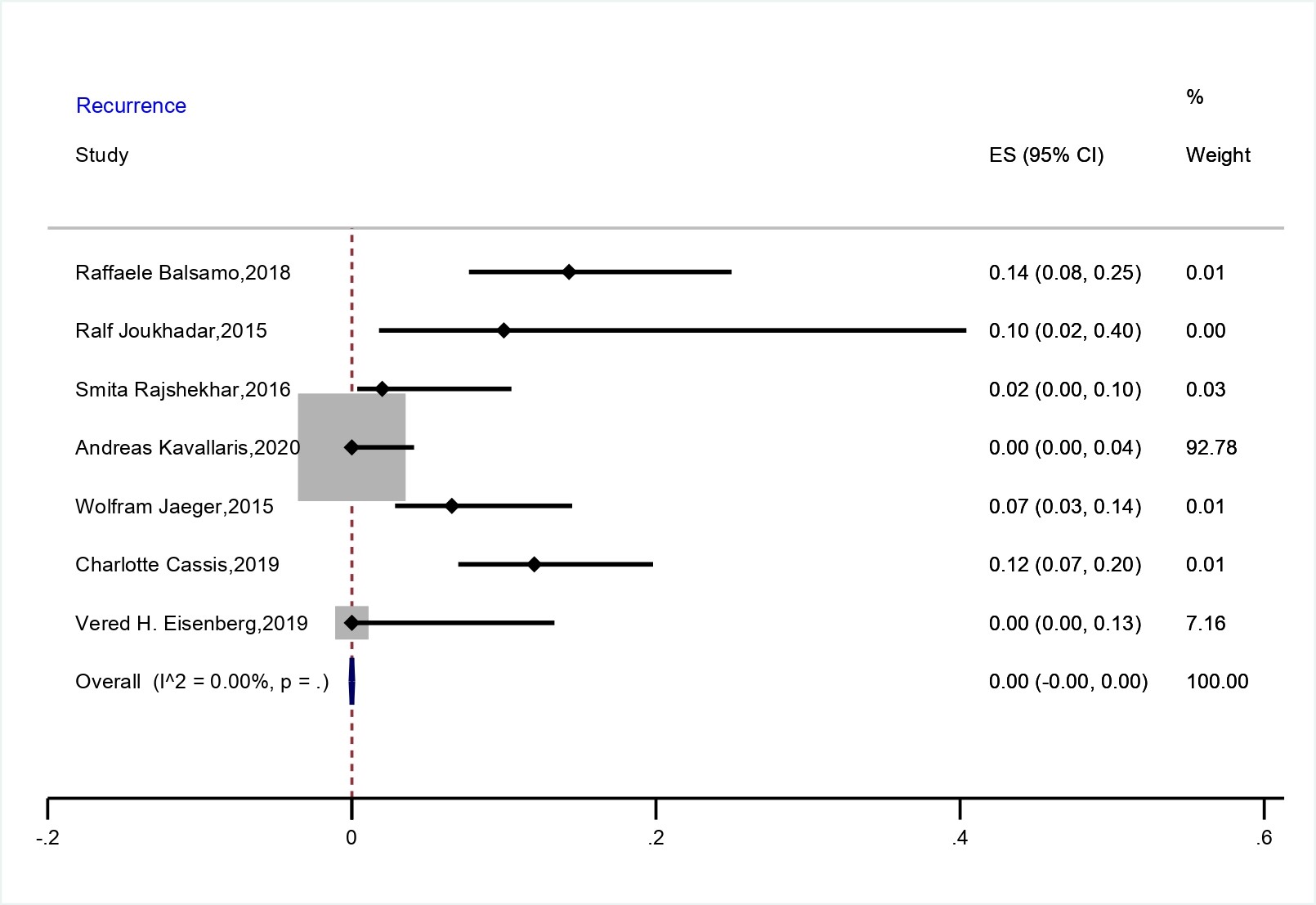

Supplement: Supplementary file 1 — Supporting Information Supporting File 1 (Search strings.docx): This document contains the exact search strategies applied to PubMed, Scopus, Web of Science, Embase, and Google Scholar for the systematic literature review. Figure S1. Forest plot of prevalence of mesh extrusion in PVDF patients. Figure S2. Forest plot of prevalence of prolapse recurrence in PVDF patients. Figure S3. Forest plot of prevalence of reoperation in PVDF patients. Figure S4. Forest plot of prevalence of sexual dysfunction in PVDF patients. Figure S5. Forest plot of prevalence of urge urinary incontinence in PVDF patients. Figure S6. Forest plot of prevalence of stress urinary incontinence in PVDF patients. Figure S7. Forest plot of prevalence of de novo urinary incontinence in PVDF patients. Figure S8. Funnel plot of mesh extrusion prevalence. Figure S9. Funnel plot of reoperation prevalence. [file AIU-2026-1620419-s001.zip › Fig S2.jpg]

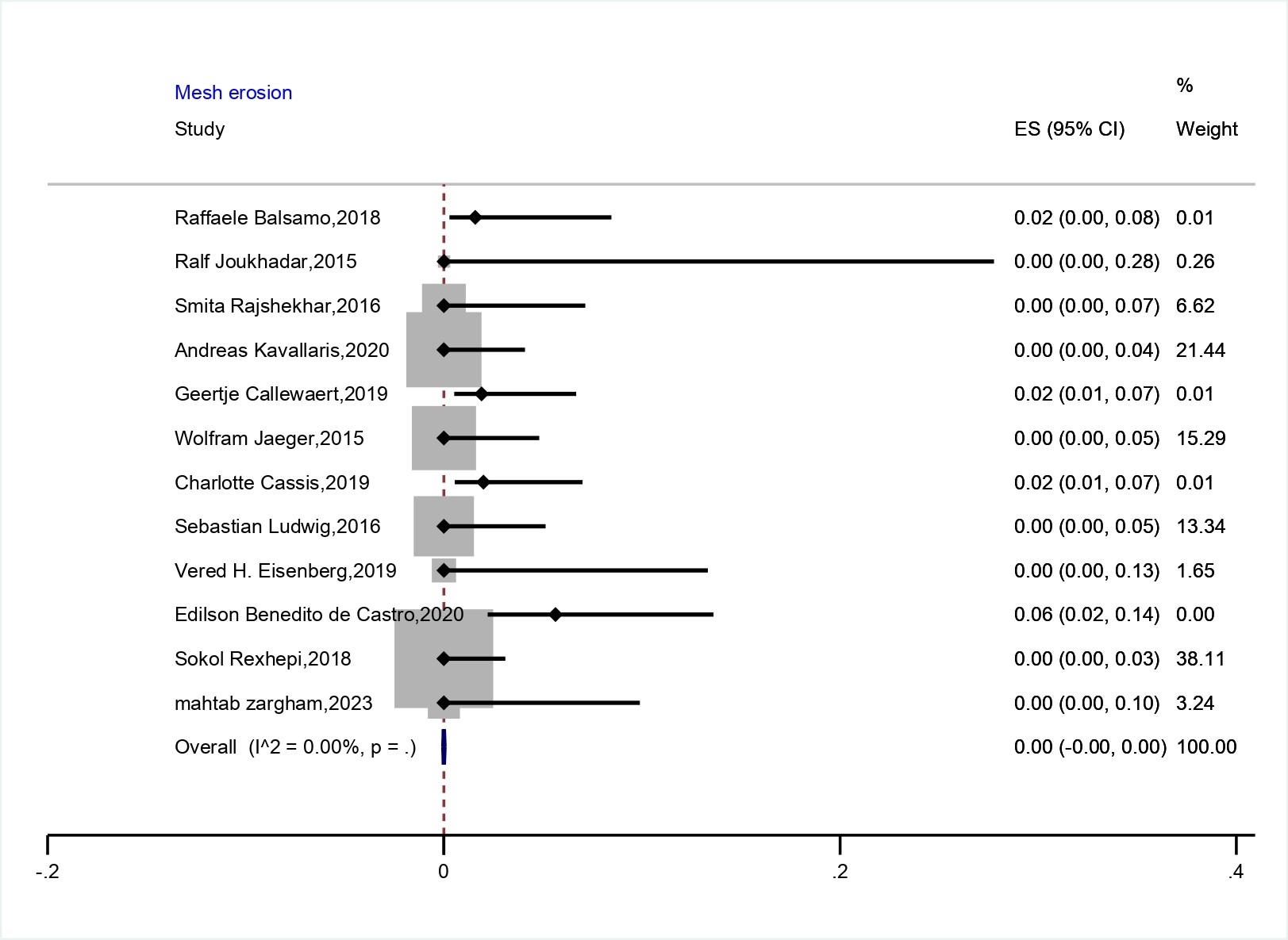

Supplement: Supplementary file 1 — Supporting Information Supporting File 1 (Search strings.docx): This document contains the exact search strategies applied to PubMed, Scopus, Web of Science, Embase, and Google Scholar for the systematic literature review. Figure S1. Forest plot of prevalence of mesh extrusion in PVDF patients. Figure S2. Forest plot of prevalence of prolapse recurrence in PVDF patients. Figure S3. Forest plot of prevalence of reoperation in PVDF patients. Figure S4. Forest plot of prevalence of sexual dysfunction in PVDF patients. Figure S5. Forest plot of prevalence of urge urinary incontinence in PVDF patients. Figure S6. Forest plot of prevalence of stress urinary incontinence in PVDF patients. Figure S7. Forest plot of prevalence of de novo urinary incontinence in PVDF patients. Figure S8. Funnel plot of mesh extrusion prevalence. Figure S9. Funnel plot of reoperation prevalence. [file AIU-2026-1620419-s001.zip › Fig. S1..jpg]

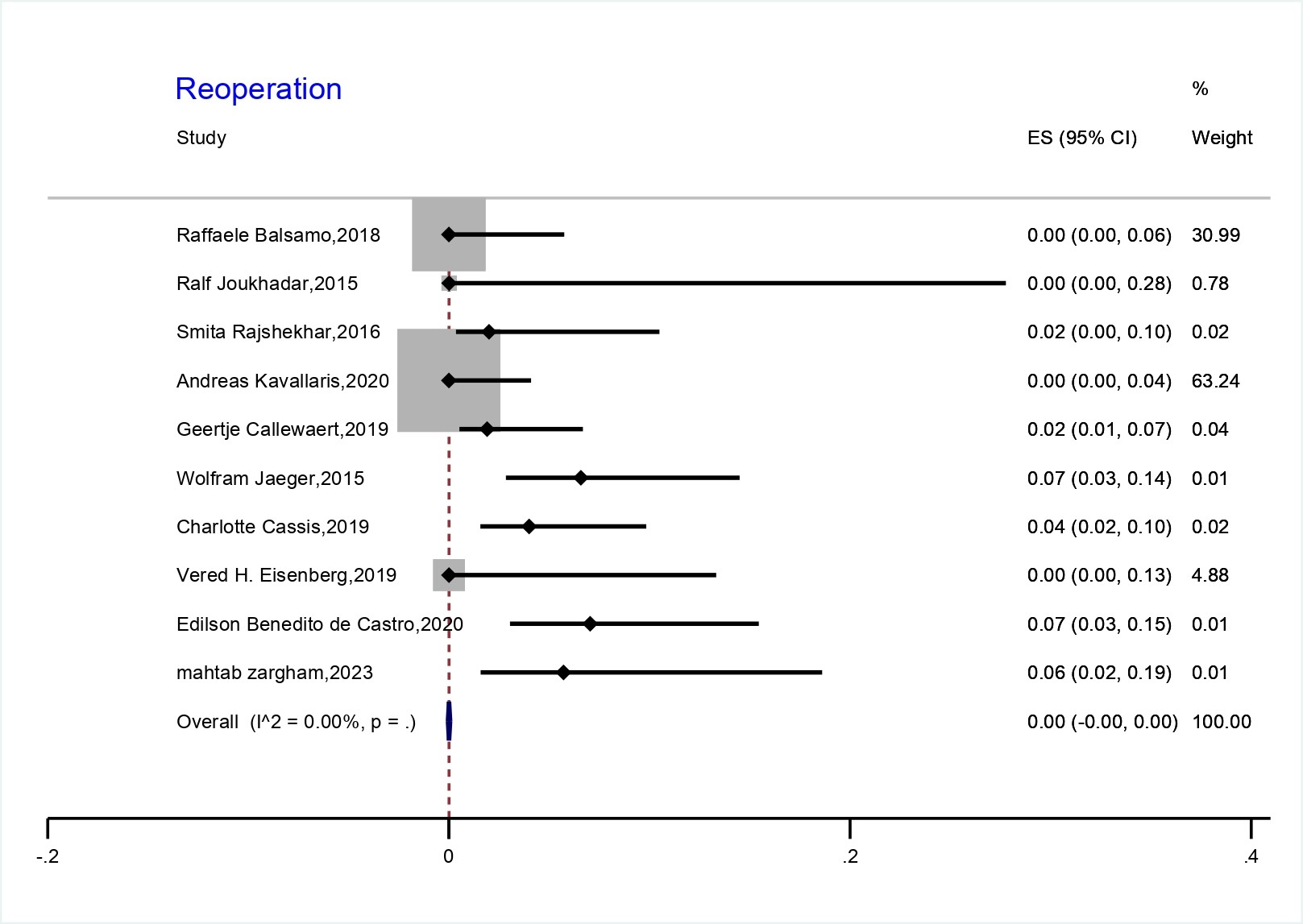

Supplement: Supplementary file 1 — Supporting Information Supporting File 1 (Search strings.docx): This document contains the exact search strategies applied to PubMed, Scopus, Web of Science, Embase, and Google Scholar for the systematic literature review. Figure S1. Forest plot of prevalence of mesh extrusion in PVDF patients. Figure S2. Forest plot of prevalence of prolapse recurrence in PVDF patients. Figure S3. Forest plot of prevalence of reoperation in PVDF patients. Figure S4. Forest plot of prevalence of sexual dysfunction in PVDF patients. Figure S5. Forest plot of prevalence of urge urinary incontinence in PVDF patients. Figure S6. Forest plot of prevalence of stress urinary incontinence in PVDF patients. Figure S7. Forest plot of prevalence of de novo urinary incontinence in PVDF patients. Figure S8. Funnel plot of mesh extrusion prevalence. Figure S9. Funnel plot of reoperation prevalence. [file AIU-2026-1620419-s001.zip › Fig. S3.jpg]

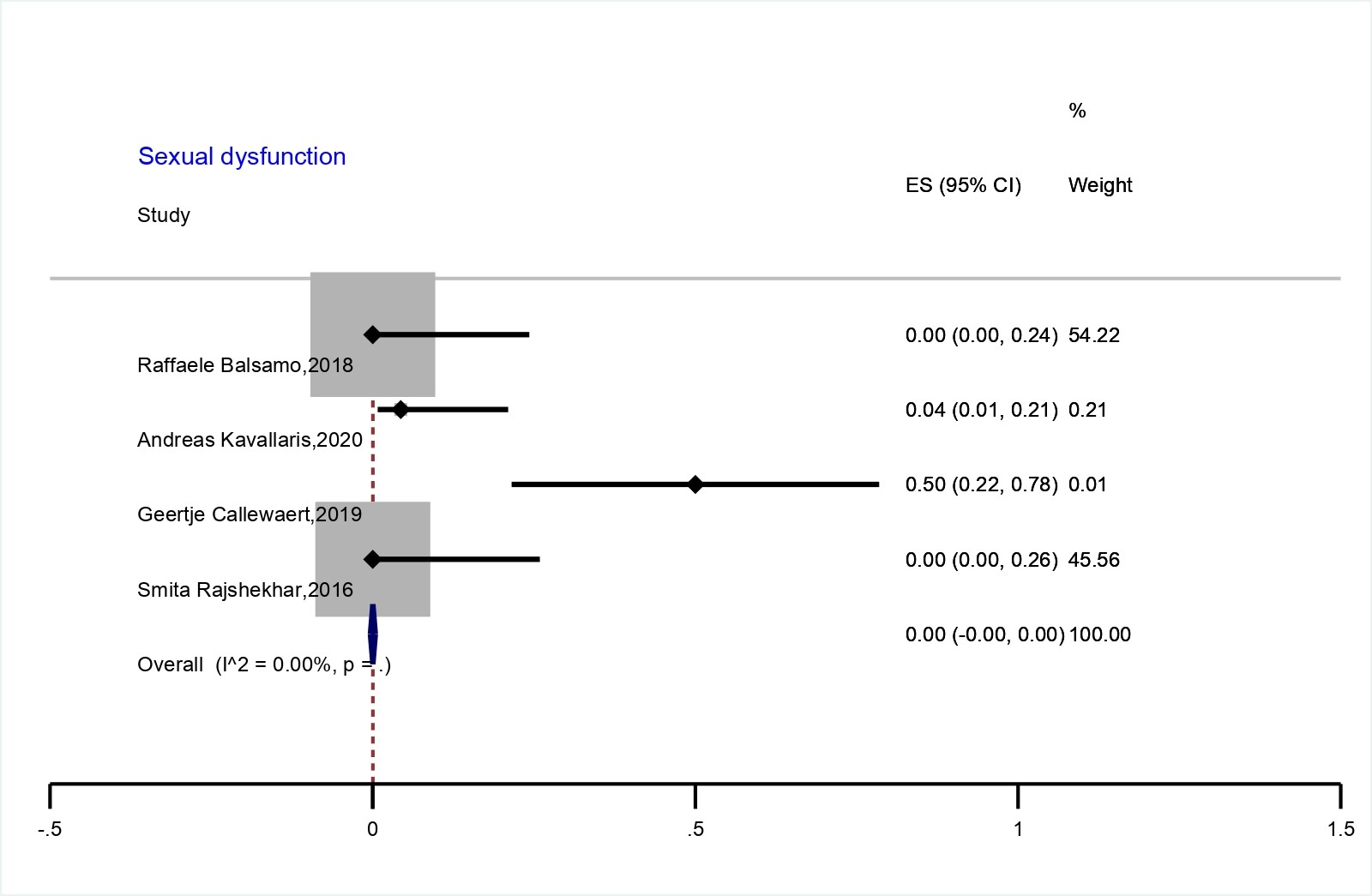

Supplement: Supplementary file 1 — Supporting Information Supporting File 1 (Search strings.docx): This document contains the exact search strategies applied to PubMed, Scopus, Web of Science, Embase, and Google Scholar for the systematic literature review. Figure S1. Forest plot of prevalence of mesh extrusion in PVDF patients. Figure S2. Forest plot of prevalence of prolapse recurrence in PVDF patients. Figure S3. Forest plot of prevalence of reoperation in PVDF patients. Figure S4. Forest plot of prevalence of sexual dysfunction in PVDF patients. Figure S5. Forest plot of prevalence of urge urinary incontinence in PVDF patients. Figure S6. Forest plot of prevalence of stress urinary incontinence in PVDF patients. Figure S7. Forest plot of prevalence of de novo urinary incontinence in PVDF patients. Figure S8. Funnel plot of mesh extrusion prevalence. Figure S9. Funnel plot of reoperation prevalence. [file AIU-2026-1620419-s001.zip › Fig. S4.jpg]

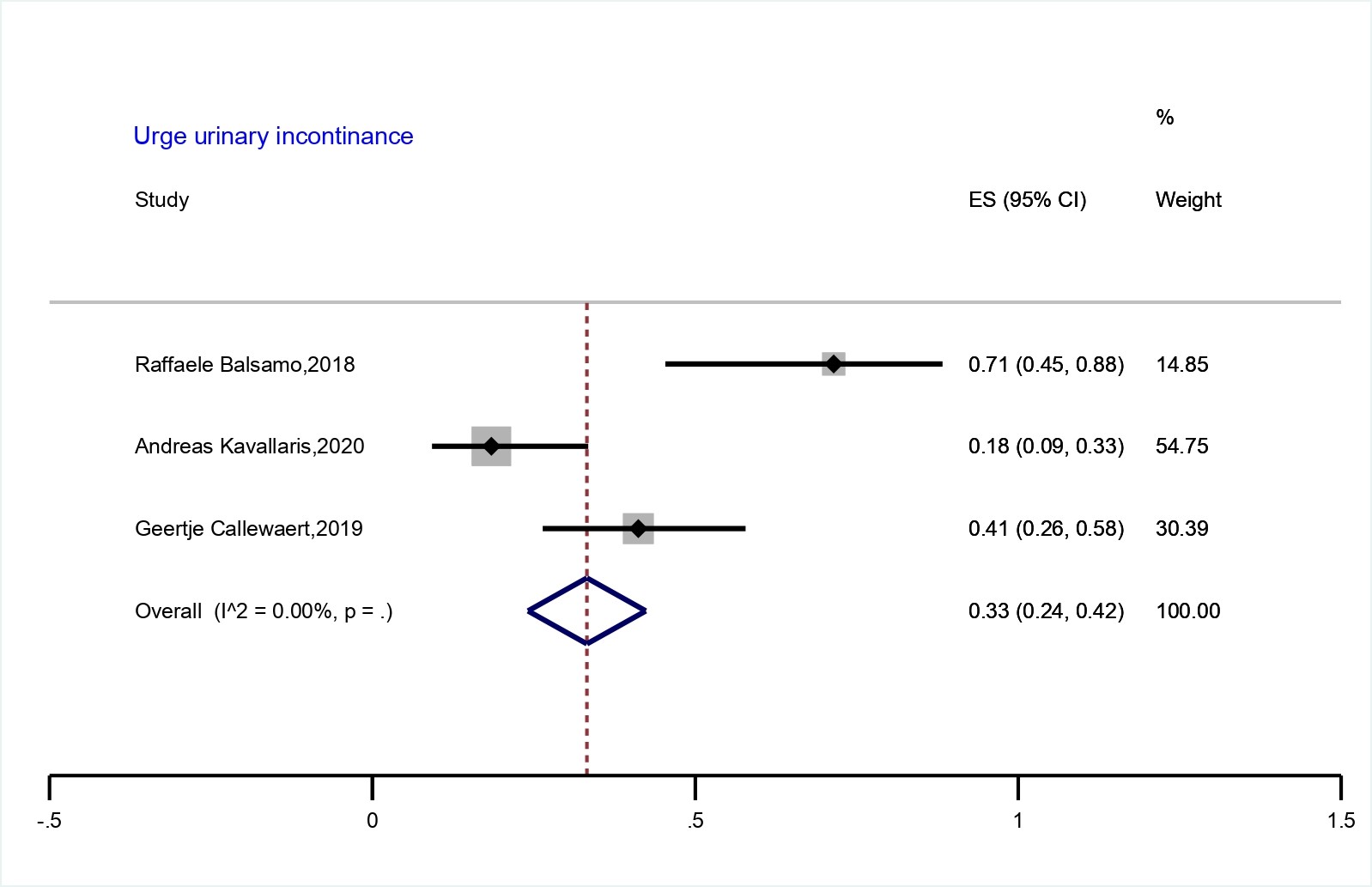

Supplement: Supplementary file 1 — Supporting Information Supporting File 1 (Search strings.docx): This document contains the exact search strategies applied to PubMed, Scopus, Web of Science, Embase, and Google Scholar for the systematic literature review. Figure S1. Forest plot of prevalence of mesh extrusion in PVDF patients. Figure S2. Forest plot of prevalence of prolapse recurrence in PVDF patients. Figure S3. Forest plot of prevalence of reoperation in PVDF patients. Figure S4. Forest plot of prevalence of sexual dysfunction in PVDF patients. Figure S5. Forest plot of prevalence of urge urinary incontinence in PVDF patients. Figure S6. Forest plot of prevalence of stress urinary incontinence in PVDF patients. Figure S7. Forest plot of prevalence of de novo urinary incontinence in PVDF patients. Figure S8. Funnel plot of mesh extrusion prevalence. Figure S9. Funnel plot of reoperation prevalence. [file AIU-2026-1620419-s001.zip › Fig. S5.jpg]

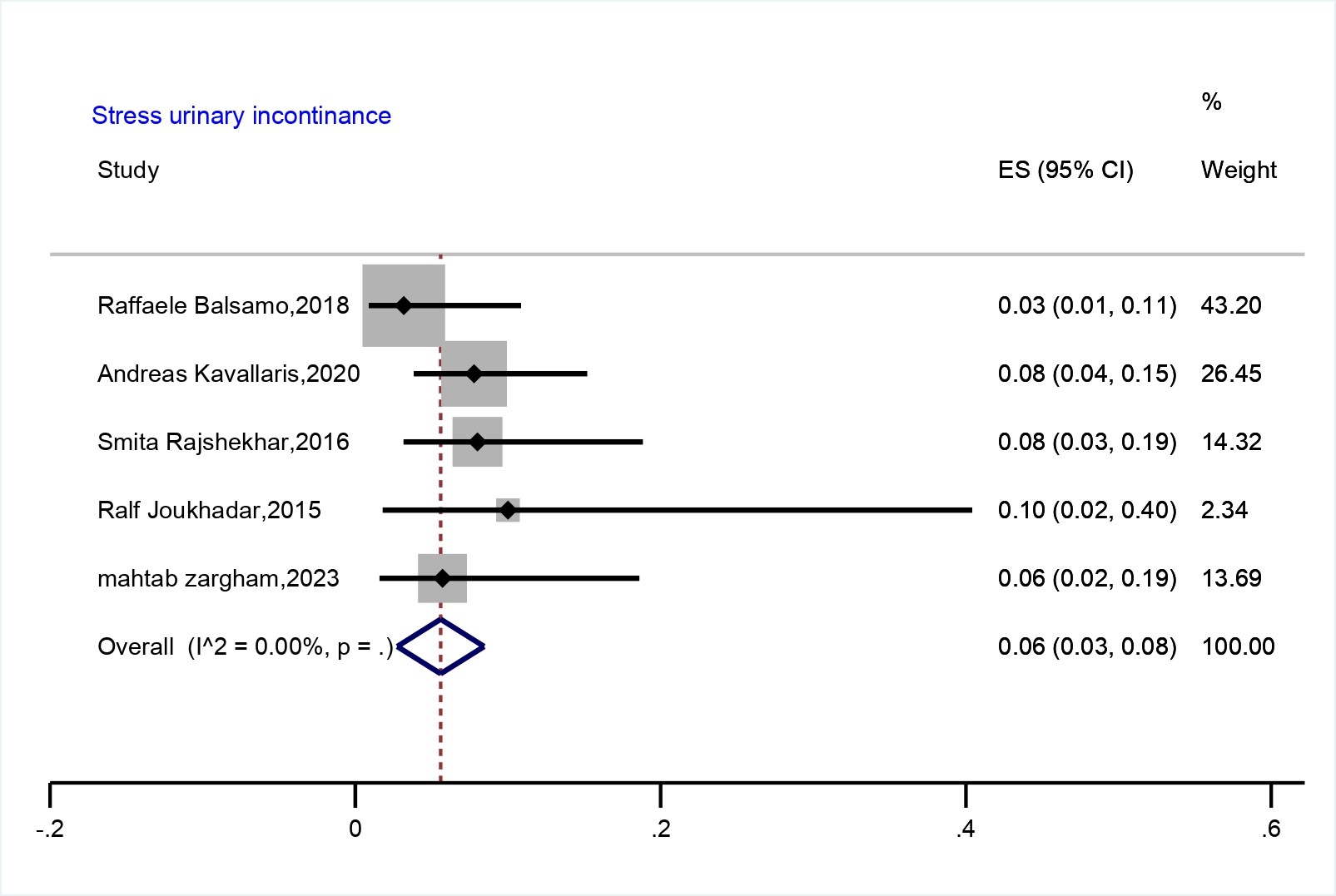

Supplement: Supplementary file 1 — Supporting Information Supporting File 1 (Search strings.docx): This document contains the exact search strategies applied to PubMed, Scopus, Web of Science, Embase, and Google Scholar for the systematic literature review. Figure S1. Forest plot of prevalence of mesh extrusion in PVDF patients. Figure S2. Forest plot of prevalence of prolapse recurrence in PVDF patients. Figure S3. Forest plot of prevalence of reoperation in PVDF patients. Figure S4. Forest plot of prevalence of sexual dysfunction in PVDF patients. Figure S5. Forest plot of prevalence of urge urinary incontinence in PVDF patients. Figure S6. Forest plot of prevalence of stress urinary incontinence in PVDF patients. Figure S7. Forest plot of prevalence of de novo urinary incontinence in PVDF patients. Figure S8. Funnel plot of mesh extrusion prevalence. Figure S9. Funnel plot of reoperation prevalence. [file AIU-2026-1620419-s001.zip › Fig. S6.jpg]

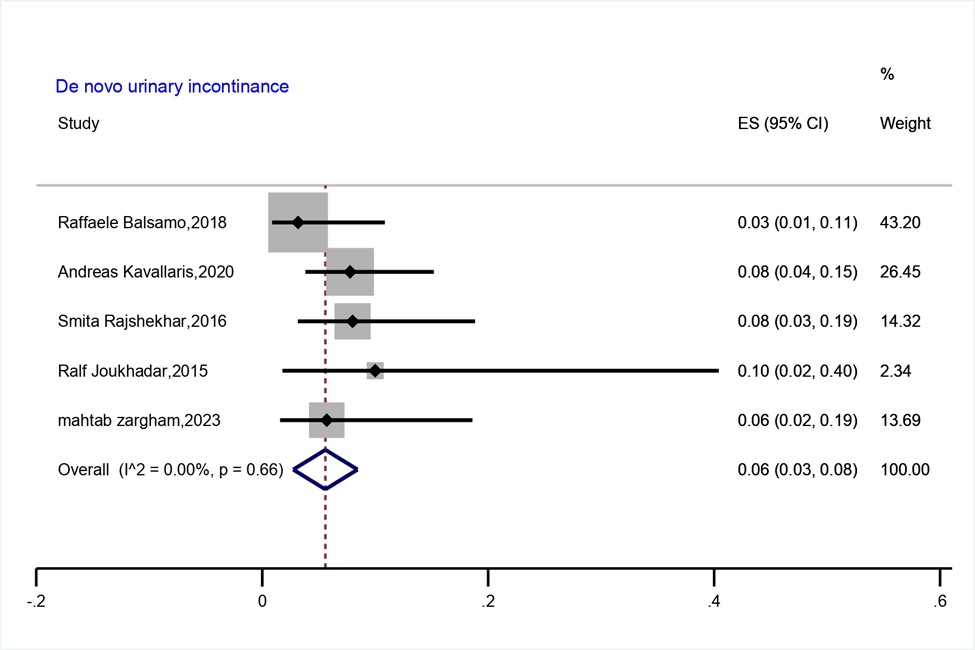

Supplement: Supplementary file 1 — Supporting Information Supporting File 1 (Search strings.docx): This document contains the exact search strategies applied to PubMed, Scopus, Web of Science, Embase, and Google Scholar for the systematic literature review. Figure S1. Forest plot of prevalence of mesh extrusion in PVDF patients. Figure S2. Forest plot of prevalence of prolapse recurrence in PVDF patients. Figure S3. Forest plot of prevalence of reoperation in PVDF patients. Figure S4. Forest plot of prevalence of sexual dysfunction in PVDF patients. Figure S5. Forest plot of prevalence of urge urinary incontinence in PVDF patients. Figure S6. Forest plot of prevalence of stress urinary incontinence in PVDF patients. Figure S7. Forest plot of prevalence of de novo urinary incontinence in PVDF patients. Figure S8. Funnel plot of mesh extrusion prevalence. Figure S9. Funnel plot of reoperation prevalence. [file AIU-2026-1620419-s001.zip › Fig. S7.jpg]

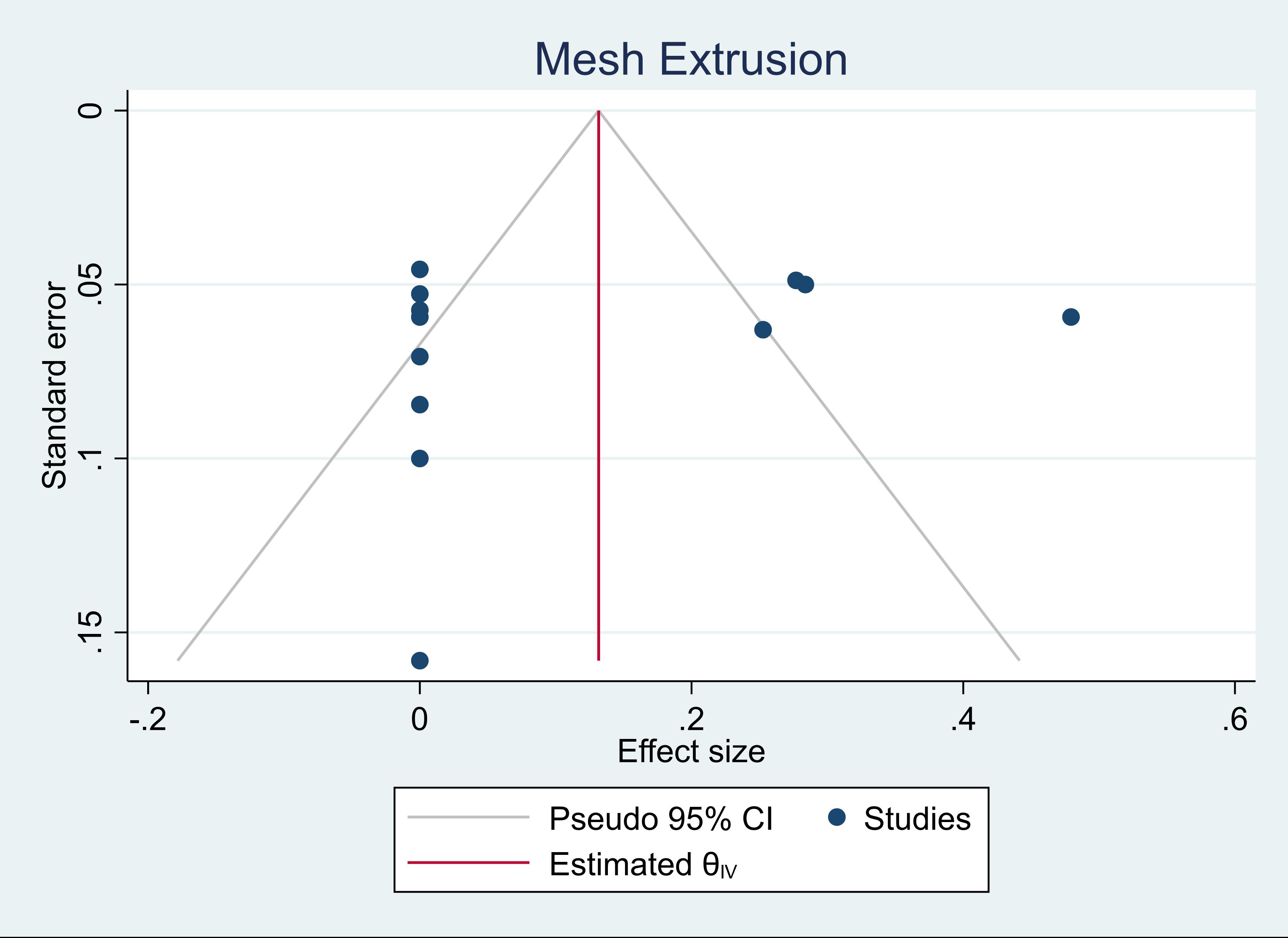

Supplement: Supplementary file 1 — Supporting Information Supporting File 1 (Search strings.docx): This document contains the exact search strategies applied to PubMed, Scopus, Web of Science, Embase, and Google Scholar for the systematic literature review. Figure S1. Forest plot of prevalence of mesh extrusion in PVDF patients. Figure S2. Forest plot of prevalence of prolapse recurrence in PVDF patients. Figure S3. Forest plot of prevalence of reoperation in PVDF patients. Figure S4. Forest plot of prevalence of sexual dysfunction in PVDF patients. Figure S5. Forest plot of prevalence of urge urinary incontinence in PVDF patients. Figure S6. Forest plot of prevalence of stress urinary incontinence in PVDF patients. Figure S7. Forest plot of prevalence of de novo urinary incontinence in PVDF patients. Figure S8. Funnel plot of mesh extrusion prevalence. Figure S9. Funnel plot of reoperation prevalence. [file AIU-2026-1620419-s001.zip › S8.2.jpg]

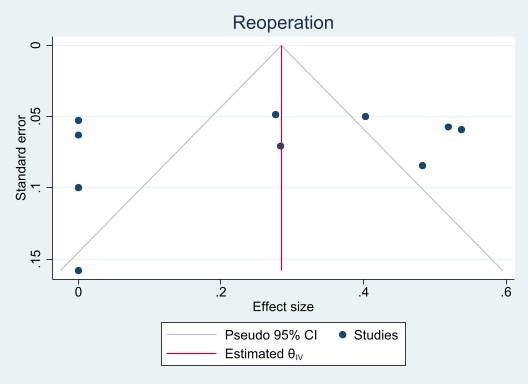

Supplement: Supplementary file 1 — Supporting Information Supporting File 1 (Search strings.docx): This document contains the exact search strategies applied to PubMed, Scopus, Web of Science, Embase, and Google Scholar for the systematic literature review. Figure S1. Forest plot of prevalence of mesh extrusion in PVDF patients. Figure S2. Forest plot of prevalence of prolapse recurrence in PVDF patients. Figure S3. Forest plot of prevalence of reoperation in PVDF patients. Figure S4. Forest plot of prevalence of sexual dysfunction in PVDF patients. Figure S5. Forest plot of prevalence of urge urinary incontinence in PVDF patients. Figure S6. Forest plot of prevalence of stress urinary incontinence in PVDF patients. Figure S7. Forest plot of prevalence of de novo urinary incontinence in PVDF patients. Figure S8. Funnel plot of mesh extrusion prevalence. Figure S9. Funnel plot of reoperation prevalence. [file AIU-2026-1620419-s001.zip › S9.1.jpg]
